# Atlanta Academy of Medicine

**Published:** 1878-01

**Authors:** J. G. Westmoreland

**Affiliations:** Reporter


### Reports of Societies.

### ATLANTA ACADEMY OF MEDICINE.

J. G. WESTMORELAND, M.D., REPORTER.

ATLANTA, Ga., December 3, 1877.

Dr. Taliaferro, President, in the chair.

Dr. Connally reported a case of uterine disease in which tympanitis was a prominent difficulty for a month or more. The abdomen is occasionally painfully disturbed and gives very clear tympanitic resonance. While this condition exists the bowels move freely without relief from the distension. Aromatics sometimes give slight relief from pain.

Dr. J. G. Westmoreland thought that gas sometimes accumulates in the cavity of the peritoneum, and that in all probability Dr. Connally's case is of this kind, since thorough evacuations do not relieve at all the tympanitic condition.

Dr. W. F. Westmoreland thought that gas in this case is most certainly in the alimentary canal. He concludes that gas does not necessarily escape with the discharge of fæces. Reasoning from analogy, thinks it never accumulates in the peritoneum, as it is not found in other cerous cavities.

Dr. Baird thought gas does not accumulate in the cavity of the peritoneum unless by decomposition, and that the case under discussion is troubled by collection in the alimentary canal.

Dr. J. T. Johnson agreed with Drs. W. F. Westmoreland and Baird, and thought gas in the cavity of the peritoneum can only exist from disorganization of the peritoneum, and that the accumulation, in the case reported, is doubtless in the alimentary canal.

Dr. Taliaferro agreed with the gentlemen last on the floor, believing that cerous membranes do not evolve gas, but that food taken into the canal is a prolific source. He thinks that females affected with chronic uterine disease are subject to paralysis of the intestinal muscular coat—perhaps from reflex depression—which leads to accumulation of gas in the canal.

Dr. J. T. Johnson mentioned the case of a young man he had seen who had a pistol shot in the temporal ridge of the right side, one inch above the angular process, passing out at the corresponding point on the opposite side. The subject exhibited no very serious symptoms from the injury. He thinks the ball must have passed through both hemispheres of the cerebrum, and considers it very remarkable that no dangerous symptoms existed.

Dr. W. F. Westmoreland, in connection, mentioned the case of a soldier shot in the forehead without alarming symptoms at once.

Dr. Johnson alluded to another case of pistol ball entering frontal bone without bad symptoms, but the subject afterward died from other injury and it was ascertained by post mortem examination that the ball in the first injury was incisted at least one inch from the surface.

Dr. Calhoun had also, several years ago, the case of a negro girl in which the temporal bone was fractured, a portion of the brain escaping through the opening. The girl was setting up and comfortable, with the loss of brain substance to the amount of a teaspoonful, and pieces of bone piercing the brain. He stitched the dura mater and closed the scalp over opening. Patient never had fever or unpleasant symptoms and came to his office in a week.

Dr. Connally mentioned a case he had ten years ago which was hurt in the evening by a blow with an axe, driving in the parietal bone of one side. The pieces were removed with portion of brain. The dura mater was closed with sutures and no bad symptoms occurred. The patient recovered promptly and without further trouble.

Dr. W. F. Westmoreland mentioned a case of necrosis from injury, in which half the cranium of a soldier was removed. Afterward he went home from the army but nothing further was heard from him.

Dr. J. G. Westmoreland reported a case of labor in which irritable uterus led to unusual delay and difficulty in delivery. A lady thirty-five years old, the mother of four children, had suffered for an hour, when he was called to see her at 1 o'clock A.M. The complaint made by the woman indicated expulsive throes and rapid progress of labor, but it was ascertained, on examination, that the uterus contracted very feebly, and that the os was not perceptibly dilated. Decided tenderness of the whole organ was manifested by slight pressure upon the abdomen and touching of the os and neck of the womb. This accounted for the great suffering of the patient at each attempt at contraction. An eighth of a grain of morphine was given to allay the irritability, and finding this ineffectual, one-sixth of a grain was given in three hours, with more effect; but as the expulsive contractions of the longitudinal fibers were still imperfect, fluid extract ergot was given, with decided benefit in this particular. Notwithstanding this increase of contractions in force and frequency, they were by no means commensurate with the suffering, and not likely to complete the delivery very soon. After fifteen hours of suffering in this way, the os being very well dilated, but the head only pressing firmly against the brim of the pelvis without any advancement, it was thought better to deliver with the forceps rather than wait longer for more favorable condition of the uterus; and with this view Dr. Taliaferro was requested to call with the instrument. On consultation, however, further test of the effects of opium in relieving the irritability, upon which the want of progress in the labor depended, was determined on, and one-fourth grain of morphine was given by the mouth, and the same amount repeated hypodermically in half an hour. In half an hour more the patient was considerably relieved from suffering, and slept a few moments. In two hours from the time a full test of opium had been determined on, effective pains came on, and in three hours the woman was delivered of a living child without further difficulty.

---

J. B. BAIRD, M.D., REPORTER PRO TEM.

---

ATLANTA, GA., December 10, 1877.

Vice-President, Dr. A. W. Calhoun, presiding.

Dr. H. L. Wilson reported the following case:

Mrs. ———, age 47, had been exercising more than usual for several days. One evening, at an entertainment, was very suddenly attacked with vertigo, causing her to stagger and fall into the arms of a friend. In the course of an hour the right side of her mouth was drawn downwards and outwards, the right eye was completely closed, with entire inability to control the lower extremities. A carriage was ordered, and she was driven to her home. Upon examination, I found that she had been menstruating irregularly for a year past. Some three or four months ago she menstruated pretty freely at one or two periods. Subsequently there has only been a slight indication of menses, accompanied with some fullness about the head, headache and considerable malaise. From the above history, I pronounced it hysterical paralysis. There being some constipation of the bowels, I ordered a mild purgative, with *hot* foot-bath. Also syrup phosphate of iron, quinine and strychnine, in twenty-drop doses, three times daily—increasing one drop until thirty-five drops were given. In addition, I applied galvanism to the spine. The next visit I found considerable difficulty in deglutition and speaking; the contraction of the face had almost disappeared upon the right side, and developed upon the left. There was considerable anæsthesia of the entire left side, with an inclination to fall to the left if she attempted to stand. In the course of a week there has been a decided improvement. She can walk with an assistant, and swallows a great deal better, talks better, etc. Her appetite has been good all the while. In addition to the phosphates, she is now taking pill of quinine, iron by hydrogen and extract belladonna. Each day shows a decided improvement, and in all probability she will soon recover her former health.

Dr. W. F. Westmoreland desired, in this connection, to mention one diagnostic symptom in this class of cases.

In the majority of instances when doubt exists, hysteria proper can be distinguished from other nervous troubles by observing that in hysterical conditions a tender spot will be found upon the spine, between the fourth and seventh dorsal vertebræ. Pressure does not always produce pain, but it shows a perverted sensibility.

Dr. Scott, of Henry county, being present, upon invitation reported a case, as follows: A man aged twenty-five years, laborer, after complaining more or less for a week, had a chill, followed by fever. The chill and fever recurred next day. For ten days after this he suffered greatly from severe headache and insomnia, though opiates were freely used. At this state, he was bled to the extent of sixteen ounces, and refreshing sleep followed. A severe nasal hemorrhage the next day was arrested by injecting into the nostril muriated tincture of iron. The pulse came down to thirty-five beats a minute, and remained so for three weeks, when it began gradually to improve, and the man had a tedious convalescence of four months' duration. In the outset of the attack his pulse was about one hundred. Consciousness was not impaired. Diarrhœa in the early stages was readily controlled. He complained of pain in the lower part of his back and in the left hypochondriac and iliac regions.

Dr. W. F. Westmoreland, in reviewing the train of symptoms, arrived at the conclusion that it was a case of typhoid fever. He could not account for the slow pulse, as there were no indications of compression or other serious brain trouble. It may be that one beat was suspended. Has encountered cases in which every other beat was omitted, due some times to organic, some times to sympathetic causes. Has known, in this way, the number of beats to be suddenly reduced one-half, and again, without warning, recover the usual number.

Dr. J. T. Johnson concurred in the opinion that the disease was typhoid fever. He thought the slow pulse was due to some idiosyncracy, and not to intermittent action. The thermometer, if it had been used, might have aided in arriving at a correct diagnosis, but even the thermometer is sometimes guilty of unaccountable freaks. He referred to a case of typhoid fever, occurring in his practice, in which the thermometer one day suddenly ran up to 107 degrees Fahrenheit.

He abandoned all hope, but the next day it indicated 93 degrees. A test of the instrument proved that it was accurate. The symptoms continued alarming for a few days, but improvement set in and recovery ensued. Has never encountered or heard of a case in which such remarkable fluctuation was noted.

Dr. Baird presented for inspection a plaster of Paris splint, for the purpose of illustrating the strength and durability of the plaster, and its advantages over other modes of dressing in certain fractures. This appliance had been used in the case of a fractured femur, and had been worn for six weeks. The mode of application was detailed several weeks ago.

Dr. Connally had seen this case several times, and was greatly pleased with the mode of dressing. He regarded the success attained complete. He reported briefly the case of a young man aged eighteen years, the victim of spinal curvature in the dorsal region. He suffered much from the superincumbent weight. With Dr. Baird's assistance he applied a plaster of Paris jacket, which afforded prompt and entire relief to his patient. Care should be observed not to leave the rollers, with the plaster, in water too long before application, as, in a few minutes the plaster will "set," when it becomes very brittle, unmanageable and inefficient.

Dr. Wilson presented to the attention of the Academy a youth aged about twelve years, the subject of chorea. About six months ago he had a severe attack of dysentery, followed by irregular movements confined to the left side of the body. One of the most remarkable features of the case is the extreme timidity of the boy, which is altogether unnatural. Tonics and general hygienic treatment, while they have improved the general health, have failed to relieve the choracic symptoms.

Dr. Baird said that doubtless a number of distinct diseases were includedd in the general term chorea; but with our present knowledge an attempt to discriminate between them is practically useless. In many cases terminating fatally, no changes are discovered in the nervous centres, while in others pathological alterations, both in the brain and the spinal cord, have been found. The fact that the disease is confined to one lateral half of the body, as occurs in a certain proportion of cases, is due to the suspension of the progress of the disease at that point of its development. The prognosis in this case he thought was favorable. As to the treatment, he advised the ether spray to the spine, from the occiput to the sacrum, every day or two, for ten or fifteen minutes, and the administration of strychnia, in gradually increasing doses, until its physiological effects are obtained, when its use should be suspended for a few days, and the same process repeated ever and anon. This, in conjunction with warm clothing, nutritious, easily digestible diet, bathing, horseback exercise, and general hygienic management, with the bromide of potassium at night, to render the sleep sound, will accomplish the end desired.

---

Atlanta, Ga., December 17, 1877.

Dr. Calhoun presented a specimen of black cataract, extracted from a man fifty-two years old. At first sight, the cataract was not detected, the pupil looking dark as usual. Close inspection, however, detected the black opaque lens.

The dark color is caused by the deposit of hæmatine, and is a very rare disease. It is the only case he had seen. The prognosis is not favorable in such cases, owing to the disease of the choroid coat which generally exists, and which is found in the case mentioned.

Dr. Baird reported a case of piles, with slight leucorrhœa, in a girl of five years old, the result of worms in the rectum. Santonine and calomel being used, a large quantity of ascarades and some lumbrocoides were discharged, after which no further trouble was experienced.

Dr. J. G. Westmoreland reported the case of a man sixty-five years old, in which a somewhat serious operation was performed from an error in diagnosis, and yet relief was afforded by it. He was called on Friday evening to see the old man on account of a reported difficulty connected with the bladder, but on examination, found a tumor in the right inguinal region, resembling exactly that made by a hernial protrusion. The history given showed that for several months a tumor similar to the one present occasionally appeared at this point while laboring, and that the recumbent posture readily caused it to disappear. For three weeks he had been quite sick, a part of the time confined to bed, but the cause of which seemed not to be known. A few days ago he came to the house, after taking a walk, suffering with the tumor as he had done frequently before, but on lying down it did not as usual disappear, the pain and soreness continuing up to the time of Dr. W.'s visit. The tumor was located exactly over and longitudinally in the direction of the inguinal canal. For two days after the tumor became permanent the bowels were not evacuated, but had been moved three times more recently. Pain was constantly present, with pretty regular paroxysms of increase every five or ten minutes. The patient, though feeble, was able to be raised for a short time; pulse about eighty. The history of the case and general appearance of the tumor decidedly favored the idea of hernia and a strong suspicion of strangulation. After an unsuccessful attempt to return the supposed contents, or even to lessen the size of the tumor, the evidences of prostration increasing the probability of strangulation, opening of the tumor was determined upon. The skin and other tissues were cut through as usual for hernia, but instead of attenuated structures, as generally found under such circumstances, they were greatly thickened, so that an incision four times the usual depth was necessary to reach the canal. When the canal was penetrated the expected bowel did not appear, but a jet of thin pus escaped, and by pressure after the opening was enlarged, not more than an ounce was discharged. The finger could readily be passed up the canal without coming in contact with anything except the spermatic cord, the deceptive elasticity of the tumor—which had not been at all reduced in size—depending, not upon an inflated bowel, but upon the swollen tissues externally. The opening was closed by sutures in the skin, and after the patient's position was changed so as to make the groin more pendent, a discharge of watery pus commenced, and was continued as position favored till perhaps two pints had escaped. The patient seemed very feeble the next day, the pulse 110, but now, three days since the operation, has good appetite, pulse 75, and seems to be doing well.

Dr. J. T. Johnson was in doubt as to the origin of the abscess, whether connected with the spine or the cœcum, but was inclined to favor the latter, as from that point the matter would be more likely to find its way into the inguinal canal.

Dr. W. F. Westmoreland could more readily decide as to the location of the abscess by the appearance of the pus and the length of time that had elapsed since the trouble commenced. If disease of the spine or any other bony structure was the cause, the pus will be thin with shreds intermixed, and would require several months to develop the present condition. On the other hand, the pus would be thicker, and the time necessary to complete the abscess only a few weeks, if connected with the cœcum.

Dr. H. L. Wilson reported a case of diphtheria in which he found satisfactory results from the local application of carbolic acid in the strength of one fluidrachm to the ounce, and internally in the dose of 3 grs. ter die for an adult.

Dr. J. G. Westmoreland reported, also, the case of a boy three years old, with stricture of the œsophagus from swallowing caustic potash. The injury occurred four months ago and the child has not been able to swallow solid food since, being nourished almost entirely by sweet milk and sugar. The stricture is near the lower part of the gullet, and will not admit a bougie larger than a crow quill. The boy's appearance proves the nourishing qualities of the articles of food mentioned, as he is stout, fat and healthy. The treatment consists of the introduction, two or three times a week, and sometimes more frequently, of a small gum catheter, into the strictured portion, but without very perceptibly increasing the size.

Dr. W. F. Westmoreland expressed an unfavorable prognosis, and could suggest no other treatment more promising than that already instituted. His only hope is in the changes which may occur in growth of one so young.

